# Epidemiology of complementary and alternative medicine therapy use in allogeneic hematopoietic stem cell transplant survivorship patients in Australia

**DOI:** 10.1002/cam4.889

**Published:** 2016-10-27

**Authors:** Julian Lindsay, Masrura Kabir, Nicole Gilroy, Gemma Dyer, Lisa Brice, John Moore, Matthew Greenwood, Mark Hertzberg, David Gottlieb, Stephen R. Larsen, Megan Hogg, Louisa Brown, Gillian Huang, Jeff Tan, Christopher Ward, Ian Kerridge

**Affiliations:** ^1^ Department of HaematologyRoyal North Shore HospitalSydneyNew South WalesAustralia; ^2^ Westmead HospitalSydneyNew South WalesAustralia; ^3^ Cancer Institute NSWSydneyNew South WalesAustralia; ^4^ Department of HaematologySt Vincents HospitalSydneyNew South WalesAustralia; ^5^ Northern Blood Research CentreKolling InstituteUniversity of SydneySydneyNew South WalesAustralia; ^6^ Department of HaematologyPrince of Wales HospitalSydneyNew South WalesAustralia; ^7^ Institute of HaematologyRoyal Prince Alfred HospitalSydneyNew South WalesAustralia; ^8^ Department of HaematologyCalvary Mater NewcastleSydneyNew South WalesAustralia; ^9^ Northern Clinical SchoolFaculty of MedicineUniversity of SydneySydneyNew South WalesAustralia

**Keywords:** Blood and marrow transplantation (BMT), complementary and alternative medicine (CAM), hematopoietic stem cell transplantation (HSCT), quality of life, survivors

## Abstract

In addition to prescribed conventional medicines, many allogeneic hematopoietic stem cell transplant (HSCT) survivors also use complementary and alternative medical therapies (CAM), however, the frequency and types of CAMs used by allogeneic HSCT survivors remain unclear. Study participants were adults who had undergone an allogeneic HSCT between 1st January 2000 and 31st December 2012. Participants completed a 402‐item questionnaire regarding the use of CAM, medical complications, specialist referrals, medications and therapies, infections, vaccinations, cancer screening, lifestyle, and occupational issues and relationship status following stem cell transplantation. A total of 1475 allogeneic HSCT were performed in the study period. Of the 669 recipients known to be alive at study sampling, 583 were contactable and were sent study packs. Of 432 participants who returned the completed survey (66% of total eligible, 76% of those contacted), 239 (54.1%) HSCT survivors used at least one form of CAM. These included dietary modification (13.6%), vitamin therapy (30%), spiritual or mind–body therapy (17.2%), herbal supplements (13.5%), manipulative and body‐based therapies (26%), Chinese medicine (3.5%), reiki (3%), and homeopathy (3%). These results definitively demonstrate that a large proportion of HSCT survivors are using one or more form of CAM therapy. Given the potential benefits demonstrated by small studies of specific CAM therapies in this patient group, as well as clearly documented therapies with no benefit or even toxicity, this result shows there is a large unmet need for additional studies to ascertain efficacy and safety of CAM therapies in this growing population.

## Introduction

Worldwide, 90,000 1 allogeneic HSCTs were performed between 2001 and 2011, with 4369 2 of these procedures occurring in Australia. With improvements in donor selection, conditioning therapies, and supportive care, 35–80% of HSCT recipients can now be expected to become long‐term survivors and be cured of their underlying disease 3. While HSCT provides a clear benefit for many patients with malignant and nonmalignant disease, it is also associated with significant morbidity and mortality 4.

In recent years, there has been growing literature on the long‐term (or late) psychosocial and medical complications experienced by HSCT survivors. The impact of these is profound with HSCT survivors experiencing a 30% lower life expectancy than a matched population cohort 4. While many survivors rate their quality of life highly at 2 years posttransplant, many HSCT recipients experience considerable difficulty coping with the short‐, medium‐, and long‐term physical and psychological sequelae of HSCT and with the uncertainties of their prognosis. Given the extent and impact of late complications of HSCT, ongoing long‐term follow‐up and multidisciplinary care of HSCT recipients is essential 5.

In addition to prescribed conventional medicines, many HSCT survivors also use complementary and alternative medical therapies (CAM). However, the prevalence and extent of the usage of CAMs in this patient group remains unclear. A review published in 1998 found the prevalence of CAM use among cancer patients ranges from 7% to 64% of patients sampled in 26 studies conducted worldwide 6. This is consistent with CAM use in the general population, estimated at 40% in Australia, Canada, Europe, New Zealand, and the United States 7.

Studies in the United States have reported that the most common complementary practices and products used by individuals with cancer are vitamin/mineral supplements, prayer for self, intercessory prayer, chiropractic/osteopathic manipulation, and herbal therapies 8. To date, no studies have reported CAM usage by HSCT patients.

Despite the lack of literature on the epidemiology of CAM use in HSCT patients, there have been a number of small trials, including randomized controlled studies showing a potential benefit for some CAM therapies such as mind and body interventions, although the majority of CAM treatments show inconclusive mixed results 9. The authors of a recent literature review of these known studies of CAMs in HSCT proposed that a current barrier to the use and research in CAM therapy is the recognition and acceptance of CAM use in this population and that epidemiologic estimates were required 9. The aim of this study was to describe the frequency and types of CAM used by HSCT survivors, with the intention of enhancing recognition of CAM use.

## Methods

### Patients and procedures

Study participants were eligible if they were >18 years of age and had undergone an allogeneic HSCT between 1st January 2000 and 31st December 2012 in New South Wales (NSW) (NSW is Australia's most populous state – with a population of ~7.5 million 10), and could read and write English. Potential participants were identified from the transplant databases of all allogeneic transplant centers in NSW, with names and phone numbers provided to the research team. Consenting participants were given the option to self‐complete the questionnaire or to complete it via a phone interview with one of the researchers. A second round of telephone calls was made to consenting participants who had not returned the survey within a month. The study protocol was approved by the Northern Sydney Coast Human Research Ethics Committee (NSLHD Reference: 1207–217M).

### Instruments

The Sydney Post HSCT Study survey (SPBS) was developed by the research team. Item construction was informed by a review of the literature and discussions with patients attending HSCT long‐term follow‐up clinics. It consisted of 402 questions, including questions relating to the use of CAMs, specifically: Nutrition and Dietary approaches, Herbal supplements, Vitamin therapies, Mind–Body therapies (e.g., Meditation), Manipulative and Body therapies (e.g., Acupuncture), Traditional medicine (e.g., Traditional Chinese Medicine), Energy medicine (e.g., Reiki), and Homeopathy. Other relevant domains included demographic data, medical complications, specialist referrals, tests and assessments, medications and therapies, infections, vaccinations, cancer screening, close personal contacts, lifestyle, occupation, and relationship status following stem cell transplantation. The questionnaire used tick box response, short answer questions, and 5‐step Likert scales measuring attitudes and other factors, and takes approximately 1 h to complete. The questionnaire was piloted in clinic and phone interviews to assess face and content validity and to check for comprehension of the survey questions.

An additional one page HSCT clinical data form (The Sydney Post HSCT Clinical Data Form) was used to collect information from the transplant database including date of transplant, date of diagnosis, stage at transplant, transplant conditioning, Graft‐versus‐Host Disease (GvHD) prophylaxis, stem cell source, and donor type.

### Measures

Participants were classified as CAM users if they used at least one therapy in any of the CAM categories. CAM use was correlated with demographics, medical complications, posttransplant medical therapies, treatments and clinical variables, relationship status, and social determinants including income and occupational status. The relationship of CAM use was further explored against a range of survey instruments that measured quality of life (Functional Assessment of Cancer Therapy – Bone Marrow Transplant (FACT‐BMT Version 4), anxiety, stress, and depression (The DASS 21), chronic GVHD (The Chronic GVHD Activity Assessment – Patient Self Report (Form B) and The Lee Chronic GVHD Symptom Scale), and an assessment of life change in response to traumatic events (The Post Traumatic Growth Inventory score).

### Statistical considerations

Categorical responses were summarized using frequencies and percentages. Parametric continuous variables were summarized using means and standard deviations, and nonparametric variables using medians and interquartile ranges. The Pearson 2 test or Fishers Exact tests were used for comparative analysis of dichotomous categorical variables. Two sample comparisons of means and medians were determined using the independent t test and Wilcoxon Rank Sum tests, respectively; greater than two sample comparisons of means and medians were determined using one‐way analysis of Variance (ANOVA) and Kruskal–Wallis tests, respectively. A two‐tailed *P* < 0.05 was used as the level of statistical significance.

## Results

### Study subjects

A total of 1475 Allogeneic HSCT were performed in the study period. Of the 669 recipients known to be alive at study sampling, 583 were contactable and were sent study packs. A total of 432 (66% of total eligible, 76% of those contacted) returned the completed survey. Three percent declined participation.

### Transplantation details

Median survival time post‐HSCT was 5 years (range: 1 year 4 months–22 years). The main indication for transplantation was acute leukemia (AML/ALL) in 226 (52.3%). Remission status was reported in 406 HSCT, of which 271 (66.8%) were CR1 or CR2. Donor type was reported in 432 transplant procedures of which the majority were siblings (57.1%) and matched unrelated donors (35.8%). Peripheral blood stem cells were used in 381 (88.1%) of transplants. Myeloablative conditioning regimens were used in 216 (50.0%) and of these 103 (47.7%) employed total body irradiation (TBI). T‐cell depleting therapy was reported in 122 (28.2%). Antithymocyte globulin (ATG) accounted for 92.6% of T‐depleting modalities, with Alemtuzumab accounting for 3.3%.

### Usage of complementary and alternative medicine therapies

A total of 239 (54.2%) of HSCT survivors used at least one form of CAM, including dietary modification (13.4%), vitamin therapy (including minerals and oils) (29.3%), spiritual and mind–body therapy (17.2%), herbal supplements (13.2%), manipulative and body‐based therapies (25.4%), Chinese medicine (3.4%), reiki (3%), and homeopathy (3%) (Table 1). One hundred and seventeen (27.2%) patients used more than one form of CAM, ranging up to seven forms of CAM (Table 2).

**Table 1:** Total complementary and alternative medical therapies (CAM) usage

Overall CAM users	54.2% (239)
Dietary modification	13.6% (59)
Vitamin therapy (ex Calcium/Vit D)	27.3% (109)
Mind–body therapy (inc spiritual)	17.2% (74)
Herbal supplementation	13.5% (58)
Manipulative and body‐based therapies	26.0% (112)
Chinese medicine	3.5% (15)
Reiki	3.0% (13)
Homeopathy	3.0% (13)

**Table 2:** Total complementary and alternative medical therapies (CAM) burden

Number of CAMs	Nil	At least 1	1	2	3	4	5	6	7
Patients	43.8% (193)	54.2% (239)	26.0% (116)	14.5% (62)	6.6% (27)	4.3% (17)	0.9% (4)	0.9% (4)	0.7% (3)

Characteristics of CAM users are shown in Table 3. There was no age difference between CAM users and nonusers. Women (*P* = 0.019), people living in a major city (*P* = 0.017), those with a university education (*P* = 0.001), and those with bone disease (*P* = 0.029) were significantly more likely to use at least one CAM. When comparing pretransplant diagnosis, type of conditioning or time from transplant, cGvHD, diabetes, cardiovascular risk, thyroid problems, anxiety, and depression, no difference in CAM use was seen.

**Table 3:** Characteristics of CAM users

Variables	CAM users (%)	Nonusers (%)	*P* value	OR (CI)
Demographic				
Gender			0.019	0.63 (0.42–0.92)
Male (*n* = 244)	123/239 (51.5)	121/193 (62.7)		
Female (*n* = 188)	116/239 (48.5)	72/193 (37.3)		
Age (years)			0.73	1.06 (0.73–1.56)
<54 (*n* = 221)	124/239 (51.9)	97/193 (50.3)		
≥54 (*n* = 211)	115/239 (48.1)	96/193 (49.7)		
Postcode			0.017	1.68 (1.0–2.5)
City – metro (*n* = 305)	180/234 (76.9)	125/188 (66.5)		
Regional or remote (*n* = 117)	54/234 (23.1)	63/188 (33.5)		
Socioeconomic				
Education				
Some high school (*n* = 53), completed high school (*n* = 78), Trade/diploma (*n* = 44), Some university (*n* = 24), completed university (*n* = 126)			0.018	–
University education (*n* = 150)	98/179 (54.7)	52/146 (35.6)	0.001	2.18 (1.36–3.42)
Other (*n* = 175)	81/179 (45.3)	94/146 (64.4)		
Posttransplant income			0.43	0.85 (0.57–1.27)
Low income (*n* = 153)^^	81/230 (35.2)	72/185 (38.9)		
Middle‐high income (*n* = 262)	149/230 (64.8)	113/185 (61.1)		
Occupational status				
Full/Part time (*n* = 211)	118/220 (53.6)	93/194 (47.9)	0.24	1.25 (0.85–1.85)
Unemployed, Retired or Casual ( *n* = 203)	102/220 (46.4)	101/194 (52.1)		
Transplant factors				
Pretransplant cancer diagnosis			0.78	1.05 (0.71–1.55)
Acute leukemia (*n* = 219)	122/228 (53.5)	97/186 (52.2)		
Other (*n* = 195)	106/228 (46.5)	89/186 (47.8)		
Years since transplant	*N* = 239	*N* = 193	0.053	–
<2 years (*n* = 57)	27 (11.3)	30 (15.5)		
2 < 6 years (*n* = 199)	105 (43.9)	94 (48.7)		
6 < 10 years (*n* = 115)	64 (26.8)	51 (26.4)		
≥10 years (*n* = 61)	43 (18)	18 (9.3)		
Conditioning			0.213	–
Myeloablative (*n* = 216)	239	193		
Reduced intensity (*n* = 225)	122 (51)	88 (45.6)		
Missing (*n* = 2)	115 (48.1)	105 (54.5)		
Clinical factors				
cGvHD	*N* = 233	*N* = 192	0.39	1.23 (0.82–1.87)
Yes (*n* = 294)	166 (71.2)	128 (66.7)		
No (*n* = 131)	67 (28.8)	64 (33.3)		
Diabetes	*N* = 209	*N* = 180	0.75	0.91 (0.51– 1.6)
Yes (*n* = 56)	29 (13.9)	27 (15)		
No (*n* = 333)	180 (86.1)	153 (85)		
Thyroid	*N* = 205	*N* = 176	0.40	1.50 (0.57–3.9)
Yes (*n* = 19)	12 (5.9)	7 (4.0)		
No (*n* = 362)	193 (94.1)	169 (96.0)		
CV Risk	*N* = 219	*N* = 186	0.49	1.14 (0.77–1.70)
Yes (*n* = 175)	98 (44.7)	77 (41.4)		
No (*n* = 230)	121 (55.3)	109 (58.6)		
Self‐reported anxiety or depression	*N* = 215	*N* = 185	0.142	1.38 (0.89–2.15)
Yes (*n* = 1160	69 (32.1)	47 (25.4)		
No (*n* = 284)	146 (67.9)	138 (74.6)		
Bone disease	*N* = 214	*N* = 178	0.029	1.62 (1.04–2.52)
Yes (*n* = 121)	76 (35.5)	45 (25.3)		
No (*n* = 271)	138 (64.5)	133 (74.7)		
Skin/Mouth cancers	*N* = 215	*N* = 183	0.59	1.13 (0.71–1.85)
Yes (*n* = 94)	53 (24.7)	41 (22.4)		
No (*n* = 304)	162 (75.3)	142 (77.6)		
Other medication use				
Med group 1 (penicillin, antiviral drug, bactrim, antifungal drug)	*N* = 239	*N* = 193	0.041	0.66 (0.45–0.98)
Yes (*n* = 176)	87 (36.4)	89 (46.1)		
No (*n* = 256)	152 (63.6)	104 (53.9)		
Med group 2 (immune drug, prednisolone)	*N* = 239	*N* = 193	0.31	0.81 (0.54–1.21)
Yes (150)	78 (32.6)	72 (37.3)		
No (282)	161 (67.4)	121 (62.7)		
Med group 3 (any blood pressure drug)	*N* = 239	*N* = 193	0. 16	0.73 (0.47–1.13)
Yes (107)	53 (22.2)	186 (77.8)		
No (325)	54 (28)	139 (72)		
Med group 4 (antidepressant, any sleeping tablet, antianxiety drug)	*N* = 239	*N* = 193	0.50	1.17 (0.73–1.88)
Yes (89)	52 (21.8)	187 (78.2)		
No (343)	37 (19.2)	156 (80.8)		
Calcium	*N* = 239	*N* = 193	0.27	1.23 (0.84–1.50)
Yes (205)	119 (49.8)	86 (44.6)		
No (227)	120 (50.2)	107 (55.4)		
Vitamin D	*N* = 239	*N* = 193	0.43	1.16 (0.79 –1.7)
Yes (244)	139 (58.2)	105 (54.4)		
No (188)	10 (41.8)	88 (45.6)		
Bone strengthening drug	*N* = 239	*N* = 193	0.41	1.25 (0.73–2.14)
Yes (65)	39 (16.3)	26 (13.5)		
No (367)	200 (83.7)	167 (86.5)		
Med Group 5 (hormonal replacement)	*N* = 239	*N* = 193	0.116	1.55 (0.89–2.73)
Yes (62)	40 (16.7)	22 (11.4)		
No (370)	199 (83.3)	171 (88.6)		
Psychosocial				
Psychiatrist	*N* = 220	*N* = 181	0.99	0.99 (0.47–2.08)
Yes (*n* = 31)	17 (7.7)	14 (7.7)		
No (*n* = 370)	203 (92.3)	167 (92.3)		
Psychologist	*N* = 221	*N* = 185	0.024	1.82 (1.07–3.09)
Yes (*n* = 74)	49 (22.2)	25 (13.5)		
No (*n* = 332)	172 (77.8)	160 (86.5)		
Social worker	*N* = 221	*N* = 183	0.21	1.4 (0.79–2.67)
Yes (*n* = 51)	32 (14.5)	19 (10.4)		
No (*n* = 353)	189 (85.5)	164 (89.6)		
Dietician	*N* = 222	*N* = 185	1.00	1.0 (0.63–1.57)
Yes (*n* = 99)	54 (24.3)	45 (24.3)		
No (*n* = 308)	168 (75.7)	140 (75.7)		
Physiotherapist	*N* = 220	*N* = 183	0.010	1.8 (1.1–3.0)
Yes (*n* = 97)	64 (29.1)	33 (18)		
No (*n* = 306)	156 (70.9)	150 (82)		
Exercise physiologist	*N* = 218	*N* = 182	0.067	2.0 (0.93–4.3)
Yes (*n* = 33)	23 (10.6)	10 (5.5)		
No (*n* = 367)	195 (89.4)	172 (94.5)		
Lifestyle				
BMI group	*N* = 239	*N* = 193	0.519	–
Normal (193)	112 (46.9)	81 (42)		
Obesity (66)	36 (15.1)	30 (15.5)		
Overweight (125)	66 (27.6)	59 (30.6)		
Underweight (13)	9 (3.8)	4 (2.1)		
Missing (35)	Median 24.48	Median 25.1		
Median (IQR)	(22.1–28.03)	(22.5–28.3)		
Doing exercise	*N* = 236	*N* = 191	0.076	1.45 (0.96–2.19)
Yes (*n* = 296)	172 (72.9)	124 (64.9)		
No (*n* = 131)	64 (27.1)	67 (35.1)		
>3times/Week (199)	124 (73.4)	75 (62.3)	0.049	0.60 (0.36–1.00)
<3 times /Week(90)	45 (26.6)	45 (37.5)		
FACT‐BMT total score	108.3 (89.7–120)	104.6 (90–119)	NS	–
Total lee	17.2 (8.5–31.1)	20.85 (10.3–29.9)	NS	–
Uncertainty score	13.5 (9–17)	14 (10–17)	NS	–
Factor total	58 (40–68)	50 (30–66)	0.001	–

FACT‐BMT, functional assessment of cancer therapy – bone marrow transplant, CAM, complementary and alternative medical therapies.

Additionally, patients taking antibacterial, antiviral, or antifungal treatment were significantly less likely to use CAMs (*P* = 0.041), whereas patients taking other prescription drugs, including immunosuppressant, cardiovascular, hormone replacement, or psychotropic medications, were no more or less likely to use CAMs. Patients who routinely saw a Psychologist (*P* = 0.024) or Physiotherapist (*P* = 0.010) were more likely to use CAMs, as were those who did regular exercise (*P* = 0.049). There was no significant relationship with FACT‐BMT and patients’ use of CAMs.

### Dietary modification

Fifty‐Nine (13.6%) HSCT survivors modified their diet in some way, including caloric supplementation (3; 0.7%), low calorie diet (6; 1.4%), gluten‐free diet (6; 1.4%), lactose‐free diet (3; 0.7%), probiotic usage (4; 0.9%), low carbohydrate diet (2; 0.5%), vegetarian or pescetarian diet (8; 1.8%), low cholesterol diet (4; 0.9%), and use of organic food (14; 3.2%). Women were more likely to make modifications to their diet post‐HSCT than men, with 32 (17%) of women and 27 (11%) of men (Male: OR: 0.59 [0.34–1.03]). Nine HSCT survivors consulted a dietician (2%).

Diagnosis, comorbidities (diabetes mellitus, cardiovascular disease), bone disease, and posttransplant cancer diagnosis were not significantly associated with dietary modification. Those using dietary modification were significantly more likely to be further out from their transplant date (median 6.6 years compared to 5.0 years [*P* = 0.04]) and reported significantly higher FACT‐BMT scores (Median 109, IQR 99–121), indicative of better quality of life.

### Herbal therapy

Herbal therapies were uncommonly used by HSCT survivors, with the most common therapies including Ginseng (5; 1.1%) and Garlic (3; 0.7%). Women were more likely to use herbal therapies (Male: *P* = 0.056, OR: 0.58 [0.33–1.01]) as were patients living in an urban area (Rural: *P* = 0.051, odds 2.0 [0.98–4.14]). Although not significant, patients using herbal supplements had higher odds of having a pretransplant diagnosis of AML/ALL (OR: 1.42 [0.798–2.55]), cardiovascular risk factors (OR: 1.32 [0.73–2.39], bone disease (OR: 1.7 [0.95–3.31]), and of seeing a psychiatrist (OR: 1.35 [0.49–3.70]). Those using herbal supplements had lower odds of being diabetic (OR: 0.83 [0.33–2.0]) or seeing a social worker (OR: 0.72 [0.21–1.92]) or a dietician (OR: 0.45 [0.19–1.04]). Patients were significantly less likely to take herbal supplements the more prescription medications they took (*P* = 0.004). There was no significant relationship between FACT‐BMT and patient's use of herbs.

### Vitamin therapy (including minerals and oils)

Self‐medication of vitamins (excluding Vitamin D and Calcium) was taken by 129 (29.3%) patients, including vitamin B (24; 5.4%), vitamin C (33; 7.5%), vitamin E (2; 0.5%), fish oil (28; 6.3%), magnesium (15; 3.4%), zinc (8; 1.8%), CoQ10 (3; 0.7%), and multivitamins (38; 8.6%). Calcium was taken by 211 (47.2%) patients and vitamin D by 250 (56.7%) patients (Table 4). Patients were more likely to take vitamin therapies if they had a university degree (OR: 1.36 [0.84–2.19], however, no other correlations were identified. Of the 239 patients taking vitamin supplements, the total number of supplements (supplement burden) varied, with most patients taking one (61; 51%) or two (32; 27%) supplements (Table 5).

**Table 4:** Vitamin supplementation

Total Vitamin Modification(ex Vit D/ Calcium)	Vitamin B	Vitamin C	Vitamin E	Fish oil	Magnesium	Zinc	CoQ10	Multivitamin	Calcium	Vitamin D
24.7% (109)	5.4% (24)	7.5% (33)	0.5% (2)	6.3% (28)	3.4% (15)	1.8% (8)	0.7% (3)	8.6% (38)	47.8% (211)	56.7% (250)

**Table 5:** Vitamin supplementation burden

Number of vitamin supplements per patient excl calcium +Vit D	1	2	3	4	5	6	7
	51% (61)	27% (32)	13% (16)	5% (6)	3% (4)	0	0.8% (1)

### Mind–body therapies (including spiritual healing)

Mind–body therapies were used by 74 (17.1%) patients, including meditation (45; 10.2%), hypnosis/breathing exercise (7; 1.6%), spiritual healing (9; 2.0%), yoga (8; 1.8%), and tai chi (5; 1.1%). Women were more likely to use mind–body therapies (22%) versus men (13%). Patients with university degrees were significantly more likely to use a spiritual and/or mental therapy (*P* = 0.008 OR: 2.20 [0.21–3.99]). Likewise, those who saw a Psychologist (*P* = 0.009, OR: 2.19 [1.21–3.99]), Psychiatrist (*P* = 0.015, OR: 2.62 [1.17–5.86]), Physiotherapist (*P* = 0.049, OR: 1.77[0.99–3.14]), Exercise physiologist (*P* = 0.022, OR: 2.48 [1.11–5.51]), or those who exercised more than three times per week (*P* = 0.042, OR: 0.48 [0.244–0.97]).

### Manipulative and body‐based therapies

Manipulative and body‐based therapies were used by 112 (26%) patients, including acupuncture (28; 6.3%), chiropractic (28; 6.3%), massage (64; 14%), osteopathy (9; 2%), physiotherapy (8; 1.8%), and reflexology (6; 1.4%). There was a marginal difference in genders seen with 58 (31.2%) women and 54 (22%) men using physical therapies. Those patients with university degrees were significantly more likely to use a manipulative and body‐based therapy (*P* = 0.001, OR: 2.24 [1.37–3.67]), whereas those with cGVHD also had a greater odds of use (OR: 1.38 [084 –2.26]).

### Traditional Chinese/ayurvedic medicine, reiki, and homeopathy

Traditional Chinese/ayurvedic medicine was used by 15 (3.5%) patients, of which (5.4%) were women and 5 (2.1%) were men. The patients were significantly less likely to take herbal traditional Chinese/ayuredic medicine the more prescription medications they took (*P* = 0.023). Reiki (or “energy medicine”) was used by 13 (3%) patients, of which 10 (5.4%) were women and 3 (1.2%) were men. Homeopathy was used by 13 (3.1%) patients, of which 8 (4.4%) were women and 5 (2.1%) were men.

## Discussion

In this survey of 583 allogeneic Australian HSCT survivors, over half (54.2%) used at least one CAM. The most common CAM therapies used were vitamin therapies (27.3%) and manipulative body‐based therapies (26.0%), with survivors also using mind–body therapies (17.2%), dietary modification (13.6%), and herbal supplementation (13.5%). This usage is consistent with internationally reported CAM usage in cancer patients 6, 11. The types of CAM therapies used by HSCT survivors in our study are similar to those used by cancer patients, with vitamins, manipulative body‐based therapies, and herbal supplementation used more often than other CAMs.

Few participants (3.1%) reported using homeopathy compared to other studies in cancer patients, which report usage up to 10% 12. This may reflect low rates of homeopathy usage in Australia compared to the United States, and/or the results of recent efforts by the Australian NHMRC (National Health and Medical Research Council) which released an advisory noting the absence of evidence for homeopathy and calling for restrictions on education and health insurance subsidies of homeopathy 13. At the same time it is of some concern that only half of responders reported taking calcium (47.8%) and vitamin D supplements (56.7%), given that these have shown to be beneficial in this high‐risk patient group 14.

As has been documented in other studies of CAM use, both in the general population and in cancer patients 6, 11, 12, we found a higher proportion of CAM usage by women (across all CAM subgroups), those with a university education and people living in a major city (which may be due to a lack of access to CAM in remote regions and smaller towns). We did not see any correlation between CAM usage and cGVHD or patient age. Although a cohort study using a questionnaire risks selection bias, with such a large response rate (76%) of our patient cohort of all contactable HSCT survivors over a 13‐year period, we believe selection bias is minimal and this is an accurate proportion of CAM usage.

Given the number of HSCT survivors taking CAM therapies, it is important to be aware of the potential harms CAM therapies may have, including interactions with conventional allogeneic therapies, particularly immunosuppressants and antifungals, as well as risks of manipulative therapies in patients with underlying bone disease and potential harm of overuse of vitamins 15, 16, 17. Other direct toxicity with CAM usage including diarrhea and vomiting are also a particular concern in transplant recipients as they may exacerbate concurrent gastrointestinal disease including cGVHD 16. It is also possible that by increasing polypharmacy and the pill burden experienced by allogeneic HSCT survivors (a group already taking a large number of supportive care medications), CAM therapies may also increase the likelihood of nonadherence 18. Alternatively, several randomized controlled studies, particularly mind and body interventions were found to have potential benefits in this patient group 9. For example, Takatsuka et al. demonstrated a beneficial effect of fish oil on the incidence of graft‐versus‐host disease (GVHD) in patients after HSCT 19 and two studies have found a significant positive correlation between massage therapy and a decline in anxiety and depression level in patients after HSCT 20, 21. Although our subgroup analysis did not replicate these results, with no significant differences in CGVHD or FACT‐BMT scores, the design of the study was inadequate for this purpose; rather it has demonstrated that a large proportion of HSCT recipients are using these CAMs up to 10 years posttransplant.

It is crucial that CAM usage is routinely assessed as part of HSCT long‐term follow‐up (LTFU). While the decision to use a CAM always remains the right of the patient, it is essential that this decision to do so is informed. These results definitively demonstrate a large proportion of HSCT survivors are using one or more form of CAM therapy. Given the potential benefits demonstrated by small studies of specific CAM therapies in this patient group, as well as clearly documented therapies with no benefit or even toxicity, this result shows there is a large unmet need for additional studies to ascertain efficacy and safety of CAM therapies in this growing population.

## Conflict of Interest

None declared.
